# Identification of nuclear factor-κB inhibitors in the folk herb Rhizoma Menispermi via bioactivity-based ultra-performance liquid chromatography/quadrupole time-of-flight mass spectrometry analysis

**DOI:** 10.1186/1472-6882-14-356

**Published:** 2014-09-25

**Authors:** Dan Sun, Mengge Zhou, Xuhui Ying, Binfeng Cheng, Yanqi Han, Yan Nie, Yuanyuan Hou, Gang Bai

**Affiliations:** State Key Laboratory of Medicinal Chemical Biology, College of Pharmacy and Tianjin Key Laboratory of Molecular Drug Research, Nankai University, Tianjin, 300071 China

**Keywords:** Bioactive constituent identification, NF-κB inhibitor, UPLC/Q-TOF MS, Rhizoma Menispermi, Anti-inflammation

## Abstract

**Background:**

Rhizoma Menispermi (RM) is the dried root of *Menispermum dauricum* DC, which is traditionally used to treat swelling and pain for sore throat, enteritis and rheumatic arthralgia in the clinic, but its bioactive compounds remain unclear.

**Methods:**

In this study, RM extract was administered orally to ICR mice followed by challenging with an intratracheal Pseudomonas aeruginosa suspension. Then mortality, histological features of lung, and inflammatory cytokines were evaluated. RM treatment significantly ameliorated Pseudomonas aeruginosa-induced acute lung inflammation and reduced levels of inflammatory mediators. To screen for potential anti-inflammatory constituents of the RM extract, a simple and rapid method based on ultra-performance liquid chromatography/quadrupole time-of-flight mass spectrometry (UPLC-Q/TOF MS) coupled with a luciferase reporter assay system to detect nuclear factor-κB (NF-κB) activity was established.

**Results:**

Using this system, seven potential NF-κB inhibitors were detected, including sinomenine, norsinoacutin, N-norsinoacutin-β-D-glucopyranoside, 6-O-methyl-laudanosoline-13-O-glucopyranoside, magnoflorine, laurifloline and dauricinoline. Furthermore, IL-6 and IL-8 assays confirmed the anti-inflammatory effects of these potential NF-κB inhibitors, in which norsinoacutin, 6-O-methyl-laudanosoline-13-O-glucopyranoside laurifloline, dauricinoline and N-norsinoacutin-β-D-glucopyranoside were revealed as new NF-κB inhibitors.

**Conclusion:**

This method of UPLC-Q/TOF coupled with the luciferase reporter assay system was initially applied to the study of RM and was demonstrated to represent a simple, rapid and practical approach to screen for anti-inflammatory compounds. This study provided useful results for further investigation on the anti-inflammatory mechanism of RM.

## Background

Lung inflammation occurs as a consequence of complex interactions between multiple cell types, cytokines, and mediators of the inflammatory network [1]. It can lead to cytokine production, vascular leakage, and neutrophil influx by activating the immune system, and the combined response of local immune cells mediated by cytokines, chemokines and adhesion molecules contributes to severe lung injury and mortality [2, 3]. NF-κB (nuclear factor-κB) is considered to play a central role in a variety of acute and chronic inflammatory diseases [4]. During inflammation, NF-κB cooperates with multiple upstream signaling pathways and other signaling molecules by regulating and activating the expression of multiple cytokines, chemokines and inflammatory mediators [5, 6]. Active NF-κB can enter the nucleus to activate the transcription of TNF-α (Human tumor necrosis factor alpha), IL-6 (interleukin-6), IL-8 (interleukin-8) and other inflammatory factors, which can cause the repeated phosphorylation and degradation of IκB, leading the activation of NF-κB and further increasing the expression of inflammatory cytokines [7, 8]. Thus, NF-κB is a key transcription factor in the inflammatory response, and targeting NF-κB is increasingly recognized as a fascinating opportunity to develop novel therapeutics for inflammatory disorders.

It is difficult to target the desired lesions when treating inflammatory disorders using Western drugs, such as corticosteroids and non-steroidal anti-inflammatory drugs because inflammation involves many inflammatory mediators and pathways that lead to a wide range of physiological changes [9]. Recently, traditional Chinese medicine (TCM) has drawn more and more attention because of its multi-component characteristics, including the ability to affect multiple targets and levels of signaling pathways and their multiple mechanisms to mitigate inflammation [10]. A large number of mechanistic studies have been performed by experts worldwide that demonstrate the importance and necessity of investigating TCM.

Rhizoma Menispermi (RM) is a commonly used herbal drug in TCM that has been reported to be effective for clearing away heat, removing toxic material, dispelling wind and relieving pain, and RM is typically used to treat swelling and pain for sore throat, enteritis and rheumatic arthralgia in the clinic. The dried root of *Menispermum dauricum* DC (Menispermaceae) is the predominant source of RM [11]. The primary components of RM are alkaloids, which principally could be classified as morphinane and aporphine-type alkaloids [12–14]. Presently, the ingredients magnoflorine, acutumine, acutumidin, acutuminine and sinomenine have been extracted and separated from RM [15]. The anti-inflammatory effect of a water decoction of RM on mice has been reported [16, 17]. Nevertheless, the therapeutic effect of RM on lung inflammation remains uninvestigated, and the bioactive components in RM remain unknown.

Identifying novel bioactive compounds from TCMs remains a challenge, although many of these compounds have been demonstrated to be effective based on modern pharmacological studies and clinical trials. Traditional methods based on the extraction and isolation of purified compounds to screen for bioactive compounds consume a great deal of time and sample. Therefore, a rapid and effective screening method is necessary. Ultra-performance liquid chromatography/quadrupole time-of-flight mass spectrometry (UPLC/Q-TOFMS) is being widely applied to analyze and identify TCM components due to its high peak capacity, high resolution, greater speed of analysis, and the advantages of structural information derived from TOFMS (time-of-flight mass spectrometry) technology for accurate mass determination [18, 19]. Our group has developed an approach combining UPLC/Q-TOF-MS with a luciferase reporter assay system to rapidly screen for inhibitors of NF-κB, which is a simple and effective strategy to rapidly screen for anti-inflammatory compounds in TCM preparations [20].

*Pseudomonas aeruginosa* is a ubiquitous opportunistic pathogen [21, 22]. It is a gram-negative bacterium which causes various infections, especially in patients with compromised host defense mechanisms [23, 24]. *P. aeruginosa* causes a remarkably higher mortality than other lung infection pathogens and has been categorized as one of the most pressing threats to the future of human health by the Infectious Diseases Society of America [25, 26]. *P. aeruginosa* can colonize in airway epithelium with its surface appendages such as flagella and pili. And it produces toxins such as type III secretion protein, pyocyanin and LPS, and rapidly causes serious lung inflammation. A comprehensive reaction of immune cells such as macrophages, neutrophils, and lymphocytes mediated by cytokines and chemokines can also contribute to severe lung injury and mortality. It has been shown that inflammatory cytokines and chemokines such as TNF-α, IL-1β, IL-6, IL-8, and RANTES have deleterious effects in the progression and persistence of lung inflammation [27, 28].

In this study, the anti-inflammatory effect of RM on lung inflammation induced by the *Pseudomonas aeruginosa* PAK strain was investigated. The bioactive compounds were screened using UPLC-MS and NF-κB luciferase reporter system assays. This study could indicate the potential anti-inflammatory agents of RM and provide useful results for further investigation on the anti-inflammatory mechanism of RM at the molecular level.

## Methods

### Chemicals and materials

Strain PA68 was a clinical isolate from the sputum of a patient suffering from bronchiectasis [29]. HPLC-grade acetonitrile was purchased from Merck (Darmstadt, Germany). Deionized water was purified using a Milli-Q system (Millipore Laboratory, Bedford, MA, USA). RM, Lot No. 1208076931, was purchased from AnGuo Changan Limited Company (HeBei, China) and identified by Professor Tiejun Zhang from the Tianjin Institute of Pharmaceutical Research. Magnoflorine and sinomenine were purchased from Yifang S&T (Tianjin, China). N-norsinoacutin-β-D-glucopyranoside, norsinoacutin, dauricinoline laurifloline and 6-O-methyl-laudanosoline-13-O-glucopyranoside, were isolated and purified from RM by our group, which were determined to be more than 98% pure based on HPLC. TNF-α was obtained from PeproTech (Rock Hill, NJ). Cefradine (Cef) capsules were purchased from Hainan Haili Pharmaceutical Co., Ltd (Hainan, China). Dexamethasone (Dex) was purchased from Sigma Chemical Co. (St. Louis, MO, USA). CO_2_ was purchased from Industrial Gas Distribution Co., Ltd., Tianjin hexagonal. Avertin was purchased from Kangkede Technology Co., Ltd (Tianjin, China). All reagents for cell culture were purchased from Gibco BRL Life Technologies (Rockville, MD, USA). Lipofectamine 2000 transfection reagent was obtained from Invitrogen (Carlsbad, CA, USA). All other reagents used in this study were of analytical grade.

### Animals

ICR mice (male, 18–22 g) were purchased from the Experimental Animal Center of the National Institute for the Control of Pharmaceutical and Biological Products (Beijing, China). All Animals were housed in standard conditions with a normal diet under an ambient temperature of 23–26°C and 40%-65% relative humidity with a 12 h light/dark artificial light cycle.

### Ethics statement

All experimental protocols carried out conformed to the Guide for the Care and Use of Laboratory Animal Care and Use in Research (Ministry of Health, Beijing, China), and were approved by the Animal Ethics Committee of Nankai University.

### Sample preparation

RM powder (10 g) was added to 10 volumes of water and decoated for 1 h. The decoction was filtered and then concentrated to a final concentration of 1 g/mL (crude drug/decoction).

One gram of RM was lyophilized and sequentially extracted using 10 mL of petroleum ether, dichloromethane, ethyl acetate, *n*-butanol and distilled water. Each fraction was evaporated in vacuo. Then, the petroleum ether extract (PEE), dichloromethane extract (DME), ethyl acetate extract (EAE), *n*-butanol extract (BUE) and aqueous extract (AQE) were obtained. The extracts were dissolved in a 0.1% DMSO solution and resuspended in cell-cultured medium for the luciferase activity assays.

The BUE fraction was dissolved in methanol and filtered using a 0.22 μm filter prior to LC/MS analysis.

### *P. aeruginosa*PAK strain-induced acute lung infection mouse model

An acute lung infection test was performed using the *P. aeruginosa* PAK strain-induced mouse model [21]. The mice were randomly separated into the following six groups: control group, model group; low, middle and high dose RM (RM-L, RM-M and RM-H) groups, and positive control (Cef) group. Each group consisted of 10 mice. All mice were treated with the *P. aeruginosa* PAK strain, except for the mice in the control group. The mice in the RM groups were intragastrically administered the appropriate dose (1, 3 or 9 g crude drug/kg) for 7 consecutive days. The positive control and model group mice were administered Cef (100 mg/kg) and normal saline, respectively. The *P. aeruginosa* PAK strain was cultured in Luria-Bertani medium and resuspended in sterile PBS at a concentration of 10^9^ colony-forming units/mL. Prior to the challenge, the mice were anesthetized via intraperitoneal injection of avertin (250 mg/kg). The mice were then intratracheally challenged with 40 μL of *P. aeruginosa* PAK. Whole blood and lung tissue samples were collected 24 h after stimulation. The lung tissue biopsies were fixed using 4% buffered formalin and embedded in paraffin for hematoxylin and eosin (HE) staining. For survival studies, the mice were observed regularly over the subsequent 1 d after challenged with PAK. Survival was monitored every 4 h and number of deaths in all the groups were recorded. After P. aeruginosa PAK infection, each mouse was given ad libitum access to food and water. Data was plotted on Kaplan Meir’s survival curve using Prism 5.0 software (GraphPad software Inc., San Diego, CA, USA).

### Measurement of proinflammatory cytokines

Commercially available enzyme-linked immunosorbent assay (ELISA) kits (Pierce/Endogen, Rockford, IL, USA) were used to measure the levels of IL-6, IL-8 and TNF-α in serum. The absorbance at 450 nm of each of the samples was measured using a Bio-Rad Model 680 micro-plate reader. The levels of IL-6, IL-8, and TNF-α were determined based on standard curves and expressed as pg/mL. Similarly, the levels of IL-6 and IL-8 in supernatant were measured via this method.

### Cell culture and transfection

Human embryonic kidney (HEK) 293 cells were purchased from American Type Culture Collection (Rockville, MD, USA). HEK 293 cells were grown to confluence in 96-well plates at 37°C and 5% CO_2_ in a humidified incubator using DMEM (H) medium supplemented with 10% fetal bovine serum.

HEK 293 cells were co-transfected with the NF-κB luciferase reporter plasmid PGL4.32 (Promega, WI, USA) and the Renilla luciferase reporter vector plasmid pRL-TK (Promega) at 100 and 9.6 ng per well, respectively. Transfection was performed for 24 h using Lipofectamine 2000 according to the manufacturer’s instructions. The medium was replaced with fresh serum-free medium 24 h prior to experimentation. The cells were pretreated with the drug 1 h prior to TNF-α stimulation (10 ng/mL) for 6 h.

### Dual-luciferase assay

After the cells had been stimulated, HEK 293 cells were washed, lysed, and assayed for luciferase activity using a dual-luciferase reporter assay system (Promega) according to the manufacturer’s instructions. The relative luciferase activity was calculated by normalizing the firefly luciferase activity against that of the internal control (Renilla luciferase).

### UPLC/Q-TOF MS analysis and sample preparation for activity assays

A Waters Acquity UPLC system (Waters Co., USA) equipped with a photodiode array detector (DAD) and a Waters Q/TOF Premier Mass Spectrometer with an electrospray ionization system (Waters MS Technologies, Manchester, UK) were used for sample analysis. The data acquisition was supported by MassLynx V4.1 software (Waters Co., USA). The separations were performed using a Waters Acquity UPLC BEH C_18_ column (2.1 mm × 100 mm, 1.7 μm) at 30°C. The mobile phase was acetonitrile (A)/ water containing 1% formic acid (B) at a flow rate of 0.4 mL/min, and a gradient was performed as follows: 0–3 min, A from 5% to 10%; 3–10 min, A from 10% to 20%; 10–14 min, A from 20% to 50%; 14–16 min, A from 50% to 95%. The compounds were detected via DAD scanning from 200 nm to 400 nm. The sample injection volume for analysis was 5 μL. The ESI-MS spectra were acquired in the negative and positive ion modes. The conditions for ESI-MS analysis were as follows: the capillary voltage was set at 2.5 kV, the sample cone voltage was set at 30 V, the desolvation gas flow rate was set at 600 L/h at a desolvation temperature of 300°C, the cone gas flow rate was set at 50 L/h, and the source temperature was 100°C. The Q/TOF Premier acquisition rate was 0.1 s with a 0.02 s inter-scan delay. The MS spectra are acquired from 50 to 1500 Da. Leucine-enkephalinamide acetate (LEA) was used as the lock mass (m/z 555.2931 in ESI + mode and 553.2775 in ESI- mode) at a concentration of 200 ng/mL and a flow rate of 0.2 μL/min. MS/MS analysis was performed to calculate the mass fractions of the target ions.

For sample preparation, mass spectrometry was not performed, and the fractions were collected from the outlet of the DAD into a 96-deep-well plate every 0.5 min. Then, the solvent was evaporated until dryness in a vacuum drying oven. The residues were dissolved in 100 μL of cell culture medium for further bioactivity assays.

### Statistical analysis

Statistical analysis was performed using SPSS software, and the data were expressed as the means ± SEM. Multiple comparisons were performed using ANOVA followed by Bonferroni post hoc analysis. For single comparisons, significant differences between the means were determined using Student’s *t* test. *P* < 0.05 was considered to be statistically significant.

## Results and discussion

### Effect of RM on lung infection induced by the *P. aeruginosa*PAK strain

**Figure 1:**
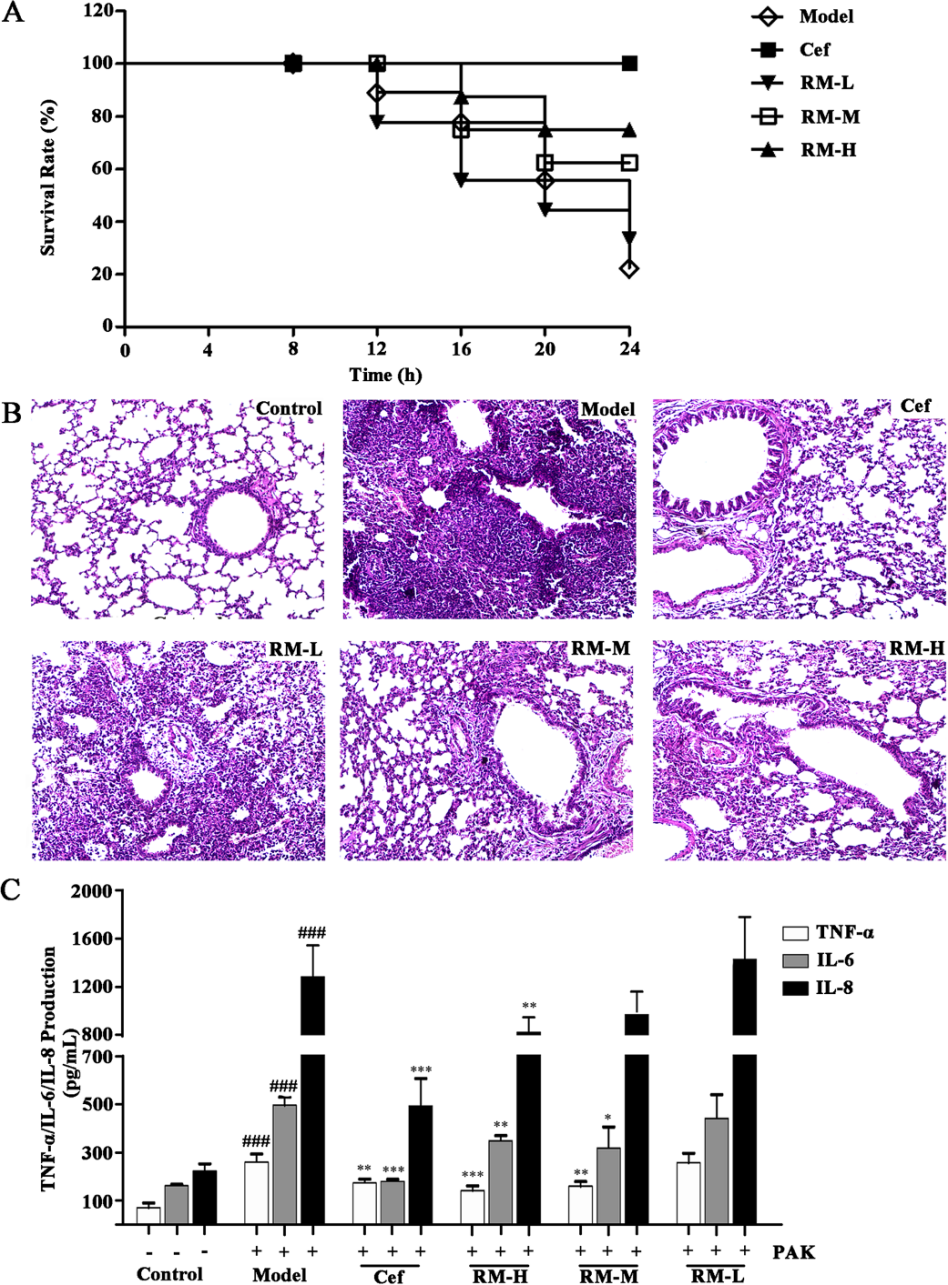
**Effect of RM on lung infection induced by the** ***P. aeruginosa*** **PAK strain.** **(A)** The survival rate of mice treated with RM or Cef upon challenge with *P. aeruginosa*. The survival rate was assessed for 24 h; **(B)** lung tissue pathology slices (the light microscopic images were captured at 100× magnification); (Control) control group, (Model) model group, (Cef) positive control group, (RM-H) high dose RM group, (RM-M) middle dose RM group,(RM-L) low dose RM group. Comparing with the control group, obvious inflammatory cell infiltration in mucosa and submucosa in model group; **(C)** effects of RM on the production of TNF-α, IL-6, IL-8 in mouse serum.Value were expressed as the mean ± SEM (*n* = 6). *p < 0.05, **p < 0.01,***p<0.001, compared to the model group (,). ^###^ *p<*0.001 compared to the control group.

The *P. aeruginosa* PAK strain was introduced to establish a mouse inflammation model. The survival rate was measured 4 h, 8 h, 12 h, 16 h, 20 h and 24 h after treatment with the *P. aeruginosa* PAK strain. The survival curve of the mice is presented in Figure 1A. Within 24 h of infection, the survival rate of the mice was only 20% in the model group, compared to 30%, 70% and 80% in the low, middle and high dose RM groups, respectively. No death was observed in the positive control group. These results suggest that RM exerts a protective effect against *P. aeruginosa* PAK strain lung infection.Pathologic sections of lung tissue are presented in Figure 1B. Compared to the structural integrity of the lung tissue from the control group, the lung sections from the model group displayed a widened alveolar septum, capillary congestion, and inflammatory cell infiltration around the plasma vessels. This result indicated that the infection model was successfully established. The inflammatory infiltrates were significantly alleviated in the high dose RM group, with less neutrophil recruitment and reduced histological injury. Histopathological evaluation of inflammation revealed that RM exerted anti-inflammatory effects on the mouse lung infection model.

Furthermore, the levels of the inflammatory cytokines IL-6, IL-8 and TNF-α in serum samples from the mice were determined via ELISA. As shown in Figure 1C, the levels of IL-6, IL-8 and TNF-α in the model group were significantly higher (more than control) (*p* < 0.001). Compared to the model group, the levels of IL-6, IL-8 and TNF-α were significantly decreased in both the middle and high dose RM groups (*p* < 0.001 in TNF-α, *p<*0.01 in IL-6 and IL-8 of RM-H, *p<*0.01 in TNF-α, *p<*0.05 in IL-6 of RM-M). These results demonstrated that RM treatment could strongly inhibit the induction of certain inflammatory cytokines by *P. aeruginosa*.

### Effects of the BUE of RM on NF-κB activity

**Figure 2:**
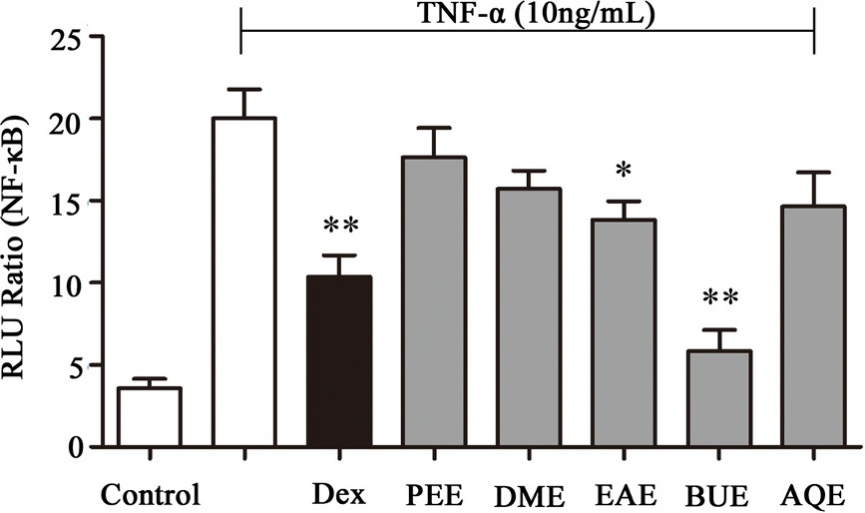
**Effects of the five extracts from RM on the level of NF-κB activity in TNF-α-stimulated HEK 293 cells.**

The effects of the different extracts of RM on TNF-α-stimulated NF-κB activity in HEK 293 cells are presented in Figure 2. Dex was used as positive control for NF-κB activity. The crude drug concentration of RM is 100 mg/mL of the various extracts. As a result, Dex (10^-5^ mol/L) and the BUE of RM significantly inhibited TNF-α-induced NF-κB production (Figure 2), indicating that the BUE extract represents a source of potential NF-κB inhibitors. Although EAE also weakly inhibited NF-κB activity, the BUE extract was used for the further identification of RM compounds via UPLC-Q/TOF MS analysis and bioactivity detection due to its greater potential for NF-κB inhibition.

### UPLC/Q-TOF MS analysis of the BUE of RM

**Figure 3:**
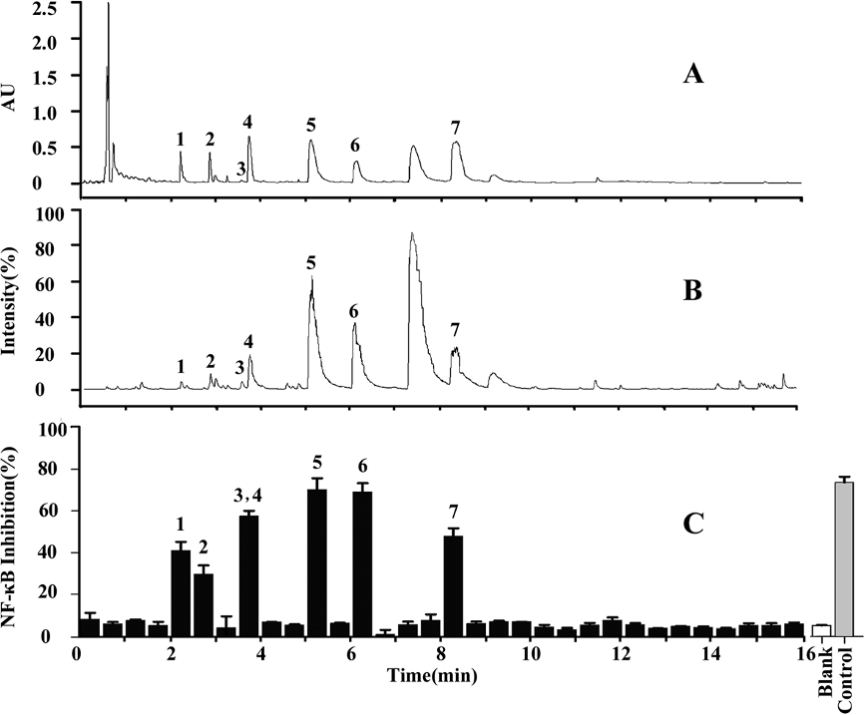
**UPLC/Q-TOF MS and bioactivity analysis of the BUE of RM. (A)** UPLC/UV chromatograms of the BUE of RM; **(B)** TIC chromatogram in the positive ESI mode; and **(C)** the chromatogram of the NF-κB activity detected via the dual-luciferase reporter assay system to determine NF-κB inhibition. The peak values are consistent with those reported in Table 1.

The optimal UPLC/Q-TOF-MS method was performed on the BUE of RM. The column effluent was split 1:9 after substance separation. One part was directed toward Q-TOF-MS analysis, and the others were directed toward diode array detection. The total ion current chromatograms in the positive ion mode are shown in Figure 3B. [M + H]^+^ ions were recorded with as much relevant information as possible to confirm the molecular weight, and the structure of all of the constituents were deduced based on their exact molecular weight, which was compared with the literature data and the natural products information reported in [30]. The MS/MS fragments and the retention time verified the conclusions. Some peaks that had the same protonated molecules in the MS spectra and similar fragment ions in the MS/MS spectra could be distinguished and identified due to their different retention behaviors. In the present study, peak 1 at 2.23 min was chosen to demonstrate the identification approach that was taken. The base peak in the positive ESI mode was *m*/*z* 476.1713, which was confirmed to be the [M + H]^+^. The elemental and possible molecular compositions (C_24_H_29_NO_9_) were deduced based on the exact molecular weight, and the molecular compositions that were retrieved from the fragmentation pathways were consistent with the literature [31]. Peak 1 was thus determined to correspond to N-norsinoacutin-β-D-glucopyranoside. Similarly, based on the fragmentation pathway and high-resolution mass to four decimal places, the other constituents were identified. The detailed results and structures of the seven candidate compounds are presented in Table 1.

**Table 1:** **The information about formula, maximum absorption wavelength, retention time, product ions and the identification results for the bioactive compounds in RM**

Peak no.	***t***_R_(min)	UV (***λ***_max_)	Identification	Composition	MW	Mode	MS ^2^
1	2.23	203,283	N-norsinoacutin-β-D -glucopyranoside	C_24_H_29_NO_9_	475.1842	Pos	476[M + H]^+^, 314[M + H-C_6_H_10_O_5_]^+^, 136[ M + H-C_6_H_10_O_5_-NC_6_H_12_O_5_]^+^
2	2.89	201,280	6-O-methyl-laudanosoline -13-O-glucopyranoside	C_24_H_31_NO_9_	477.1999	Pos	478[M + H]^+^, 316[M + H-C_6_H_10_O_5_]^+^, 194[M + H-C_6_H_10_O_5_-C_7_H_4_OHOH]^+^
3	3.60	203,251	sinomenine	C_19_H_23_NO_4_	329.1627	Pos	330[M + H]^+^, 301[M + H-CH_3_N]^+^, 271[M + H-CH_3_N-CH_2_O]^+^
4	3.77	226,286	norsinoacutin	C_18_H_19_NO_4_	313.1314	Pos	314[M + H]^+^, 299[M + H-NH]^+^, 269[M + H-NH-CH_2_O]^+^
5	5.14	224,268	magnoflorine	C_20_H_24_NO_4_	342.1700	Pos	342[M]^+^, 297[M-C_2_H_7_N]^+^, 266[M-C_2_H_7_N-CH_2_OH] ^+^
6	6.12	227,280,305	laurifloline	C_20_H_24_NO_4_	342.1700	Pos	342[M]^+^, 314[M-CO]^+^, 269[M-CO-C_2_H_7_N]^+^
7	8.39	196,232,281	dauricinoline	C_37_H_42_N_2_O_6_	610.3042	Pos	611[M + H]^+^, 580[M + H-CH_3_O]^+^ , 388[M + H-CH_3_O-C_11_H_14_ NO_2_]^+^

### Identification on NF-κB inhibitors in the BUE of RM based on bioactivity analysis

**Figure 4:**
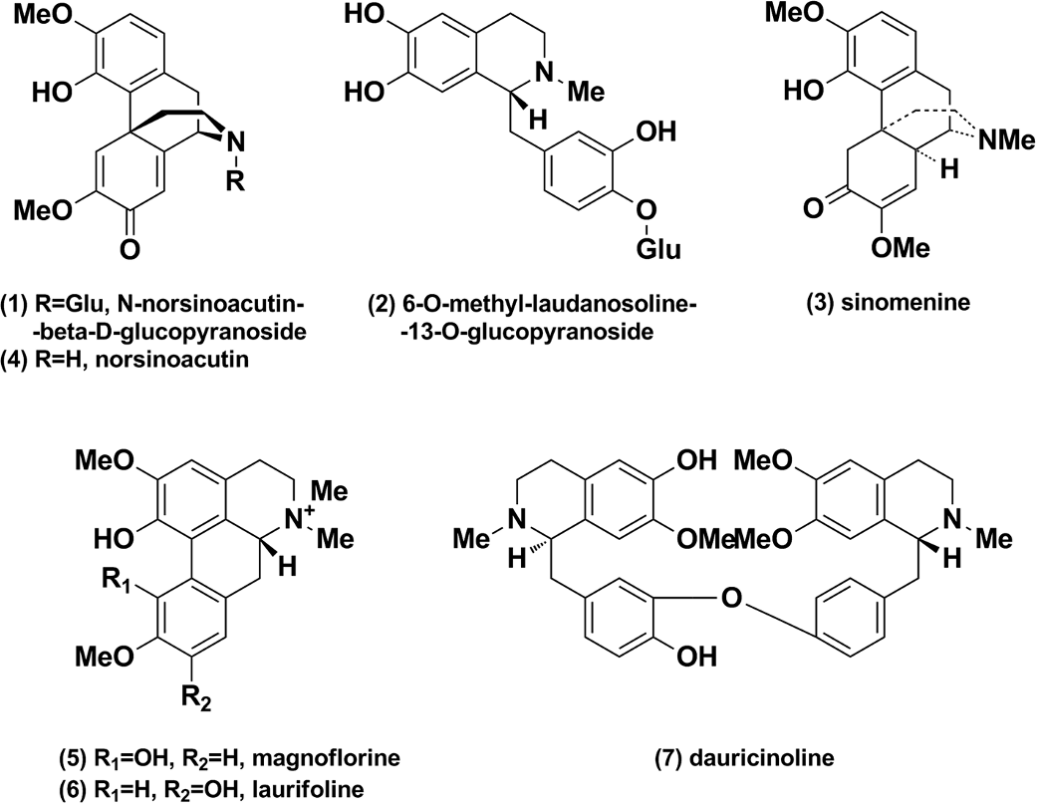
**Chemical structures of the bioactive compounds in RM.**

Following UPLC/diode array detection analysis (Figure 3A), 90% of the column effluents were collected as 0.5 min fractions for the bioactivity assay using the dual-luciferase reporter assay system. Each of the 36 fractions was evaluated for their effect on NF-κB activity (Figure 3C). Seven candidate compounds (Peaks 1 to 7) displayed potential NF-κB inhibition that was two times higher than the blank. The structures of these bioactive compounds in the BUE are presented in Figure 4. The potential NF-κB inhibitors include: sinomenine, norsinoacutin, magnoflorine, N-norsinoacutin-β-D-glucopyranoside, dauricinoline. laurifloline and 6-O-methyl-laudanosoline-13-O-glucopyranoside, There was a major peak between peaks 6 and 7, which was deduced as menisperine based on UPLC/Q-TOF MS. However, in our study it did not display an inhibitory effect on NF-κB activity.

### Confirmation of the bioactivity of the NF-κB inhibitors in RM

**Figure 5:**
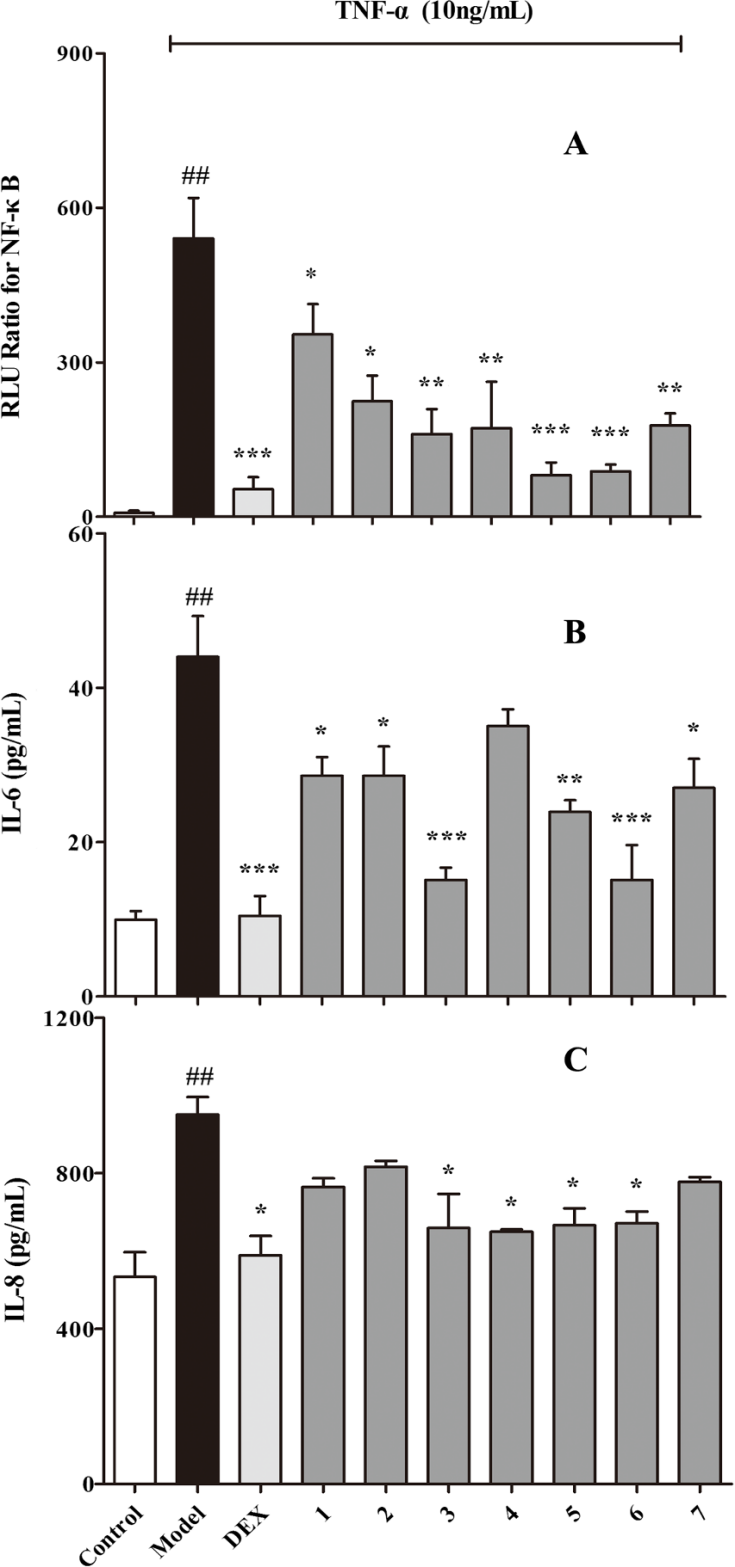
**Confirmation of the effects by potential NF-kB inhibitors.** **A**: Effects of the potential NF-kB inhibitors on the NF-kB expression. **B** and **C**: IL-6 and IL-8 expression in TNF-α induced BEAS-2B cells, respectively. Each bar represents the mean + SEM; n = 4 for each group. (1: N-norsinoacutin-β-Dglucopyranoside; 2: 6-O-methyl-laudanosoline-13-O-glucopyranoside; 3: sinomenine; 4: norsinoacutin; 5: magnoflorine; 6: laurifloline; 7: dauricinoline). *p < 0.05, **p < 0.01, ***p < 0.001 compared to the model group. ^##^ *p <* 0.01 compared to the control group.

The activities of the bioactive compounds (100 μmol/L) in RM were evaluated using the dual-luciferase reporter assay system. As shown in Figure 5A, Dex (10 μmol/L) and the seven potential NF-κB inhibitors (ingredients of RM) displayed significant NF-κB inhibitory effects (*p* < 0.05) compared to the model group. Especially, sinomenine, magnoflorine and laurifloline (*p* < 0.001) displayed highly significant inhibition. Because NF-κB transcription factors synergistically activate transcription of the inflammatory cytokines IL-6 and IL-8, the anti-inflammatory effect of these seven ingredients on IL-6 and IL-8 were further evaluated [32]. As shown in Figure 5B, IL-6 expression was reduced by six of the compounds (*p* < 0.05), highly significantly by laurifloline and sinomenine (*p* < 0.001); however, norsinoacutin displayed no significant IL-6 inhibition based on our assay. For IL-8, as shown in Figure 5C, sinomenine, norsinoacutin, magnoflorine and laurifloline demonstrated an inhibitory effect (*p* < 0.05), but the other three compounds did not display an inhibitory effect. Dex (10 μmol/L), the positive control, significantly inhibited both IL-6 (*p* < 0.001) and IL-8 expression (*p* < 0.05). The results further confirmed the anti-inflammatory effects of the seven compounds as NF-κB inhibitors in RM.

Previous studies have demonstrated that magnoflorine can increase the phagocytosis of neutrophils at a concentration of 0.5 μg/mL in *vitro*, suggesting that magnoflorine exerts significant anti-inflammatory effects [33]. Additionally, sinomenine has been used clinically to treat rheumatoid arthritis. A systematic review and meta-analysis revealed that sinomenine treatment can significantly decrease rheumatoid factor levels in patients [34]. IL-6 gene expression is also significantly diminished by treatment with 1 mmol/L sinomenine for 48 h based on RT-PCR analysis, suggesting an anti-inflammatory effect of sinomenine [35]. Consistent with a previous report [36], we demonstrated the anti-inflammatory effects of magnoflorine and sinomenine, both of which significantly reduced the expression of IL-6 and IL-8. This study is the first to report that 6-O-methyl-laudanosoline-13-O-glucopyranoside, norsinoacutin, laurifloline, dauricinoline and N-norsinoacutin-β-D-glucopyranoside inhibit the activation of NF-κB. The NF-κB protein is a transcription factor that enhances the transcription of a variety of genes, including cytokines, growth factors, adhesion molecules and immunoreceptors [37]. Upon activation, IκB is phosphorylated and degraded, leading to the activation of NF-κB, further increasing the expression of inflammatory cytokines [38, 39]. Norsinoacutin did not display significant IL-6 inhibition, and 6-O-methyl-laudanosoline-13-O-glucopyranoside, dauricinoline and N-norsinoacutin-β-D-glucopyranoside did not display an inhibitory effect on IL-8 expression in the present study. However, these compounds may act on other cytokines, which require verification in further experiments.

## Conclusions

Our results revealed that RM could contribute to the alleviation of inflammation in mice subjected to acute lung infection induced by the *P. aeruginosa* PAK strain. UPLC-Q/TOF MS coupled with a luciferase reporter assay was utilized to screen for and identification the NF-κB inhibitors that may be responsible for the anti-inflammatory effects of RM. Consequently, seven alkaloids were characterized as NF-κB inhibitors. Among these compounds, to the best of our knowledge, this is the first report that the alkaloids N-norsinoacutin-β-D-glucopyranoside, 6-O-methyl-laudanosoline-13-O-glucopyranoside, laurifloline, dauricinoline and norsinoacutin interact with NF-κB. Furthermore, IL-6 and IL-8 activity assays confirmed the anti-inflammatory effects of these seven bioactive alkaloids. This study identified the NF-κB inhibitory compounds in RM and provided useful results for further investigation of its anti-inflammatory effects at the molecular level.
